# Supplementary material for: Role of hypothalamus function in metabolic diseases and its potential mechanisms
Source: PeerJ. 2025 May 29;13:e19532. doi: 10.7717/peerj.19532 (PMC12126972; doi:10.7717/peerj.19532)
Supplement: Supplemental Information 1 [file peerj-13-19532-s001.docx]

​**​Supplementary File S1​**​.

(

(

("Hypothalamus"[Mesh] OR "Hypothalamic Nuclei"[Mesh] OR "Arcuate Nucleus of Hypothalamus"[Mesh] OR

"Hypothalamo-Hypophyseal System"[Mesh] OR "Ventromedial Hypothalamic Nucleus"[Mesh])

OR

(

"hypothalamus"[tiab] OR "hypothalamic dysfunction"[tiab] OR "neuroendocrine regulation"[tiab] OR

"energy balance"[tiab] OR "leptin-melanocortin"[tiab] OR "hypothalamic nuclei"[tiab]

)

)

AND

(

("Metabolic Diseases"[Mesh] OR "Obesity"[Mesh] OR "Diabetes Mellitus"[Mesh] OR "Diabetes Mellitus, Type 2"[Mesh] OR

"Prader-Willi Syndrome"[Mesh] OR "Bardet-Biedl Syndrome"[Mesh] OR "Monogenic Obesity"[tiab])

OR

(

"metabolic disorder*"[tiab] OR "obesity"[tiab] OR "diabetes"[tiab] OR "insulin resistance"[tiab] OR

"body weight regulation"[tiab]

)

)

AND

(

("GLP-1 Receptor Agonists"[Mesh] OR "Melanocortin-4 Receptor Agonists"[Mesh] OR "Bariatric Surgery"[Mesh] OR

"Gastric Bypass"[Mesh] OR "Sleeve Gastrectomy"[Mesh] OR "Positron-Emission Tomography"[Mesh])

OR

(

"GLP-1 agonist*"[tiab] OR "setmelanotide"[tiab] OR "MC4R agonist*"[tiab] OR "semaglutide"[tiab] OR

"liraglutide"[tiab] OR "bariatric surgery"[tiab] OR "RYGB"[tiab] OR "VSG"[tiab] OR

"extreme dietary restriction*"[tiab] OR "PET imaging"[tiab]

)

)

)

AND

("2010/01/01"[Date - Publication] : "2024/04/30"[Date - Publication])

AND

English[Language]

**Key Components Explained​​**

1. ​**​Hypothalamus Terms​**​
   - *MeSH terms*: Covers anatomical structures (e.g., "Arcuate Nucleus") and functional pathways.
   - *Free-text*: Includes variations like "hypothalamic dysfunction" and mechanistic terms (e.g., "leptin-melanocortin").
2. ​**​Metabolic Disease Terms​**​
   - *MeSH terms*: Broad categories (e.g., "Obesity," "Diabetes Mellitus") and rare syndromes (e.g., "Prader-Willi Syndrome").
   - *Free-text*: Targets pathophysiology (e.g., "insulin resistance") and niche topics (e.g., "monogenic obesity").
3. ​**​Interventions/Assays​**​
   - *MeSH terms*: Pharmacological agents (e.g., "GLP-1 Receptor Agonists"), surgical procedures (e.g., "Bariatric Surgery"), and imaging modalities.
   - *Free-text*: Specific drug names (e.g., "semaglutide"), surgery types (e.g., "VSG"), and interventions (e.g., "extreme dietary restrictions").
4. ​**​Filters​**​
   - *Date*: Limited to 2010–April 2024 for relevance to recent advancements.
   - *Language*: English only (non-English studies were excluded post-screening).
